# A first‐trimester mechanistic framework integrating three Physiopathologic biomarker domains for pre‐eclampsia classification

**DOI:** 10.1002/ijgo.70804

**Published:** 2026-01-19

**Authors:** Johnatan Torres‐Torres, Salvador Espino‐y‐Sosa, Raigam Jafet Martinez‐Portilla, Elsa Romelia Moreno‐Verduzco, Irma Eloisa Monroy‐Muñoz, Juan Mario Solis‐Paredes, Javier Perez Duran, Hector Borboa‐Olivares, Lourdes Rojas‐Zepeda

**Affiliations:** ^1^ Department of Reproductive and Perinatal Health Research Instituto Nacional de Perinatologia Isidro Espinosa de los Reyes Mexico City Mexico; ^2^ Iberoamerican Research Network in Obstetrics Gynecology and Translational Medicine Mexico City Mexico; ^3^ Department of Bioinformatics Instituto Nacional de Perinatologia Isidro Espinosa de los Reyes Mexico City Mexico; ^4^ Clinical Research Branch Instituto Nacional de Perinatologia Isidro Espinosa de los Reyes Mexico City Mexico; ^5^ Maternal‐Fetal Department Instituto Materno Infantil del Estado de Mexico Toluca Mexico

**Keywords:** angiogenic and hemodynamic domains, first‐trimester biomarkers, mechanistic classification, placental dysfunction, precision obstetrics, pre‐eclampsia

## Abstract

**Objective:**

To develop and internally validate a mechanistic, three‐domain framework for early classification and prediction of pre‐eclampsia (PE) using first‐trimester angiogenic, uteroplacental, and maternal vascular biomarkers.

**Methods:**

In a prospective cohort of 1925 singleton pregnancies screened at 11 to 13.6 weeks, placental growth factor (PGF), uterine artery pulsatility index (UtA‐PI), and mean arterial pressure (MAP) were log‐transformed and standardized to gestational age–adjusted multiples of the median. Prespecified percentile thresholds (PGF <10th; UtA‐PI >95th; MAP >95th) defined domain abnormalities and mechanistic phenotypes. Associations with PE, fetal growth restriction (FGR), and the composite of PE or FGR were assessed using logistic regression. Discrimination (area under the [receiver operating characteristic] curve [AUC]), calibration, and clinical utility were evaluated; bootstrap internal validation was used for optimism correction; and decision‐curve analysis quantified net clinical benefit.

**Results:**

PE occurred in 104 of 1925 pregnancies (5.4%). Phenotypes were distributed as normo (81.7%), molecular (7.6%), hemodynamic (3.2%), tensional (5.1%), dual (≥2 domains; 2.1%), and triple (3/3; 0.3%). The risk of PE increased stepwise from 3.9% (normo) to 80.0% (triple) (*P* for trend <0.001). The three‐domain model improved discrimination to an AUC of 0.81 (95% confidence interval [CI], 0.77–0.86) versus the clinical model (AUC, 0.68; *P* < 0.001), achieved good discrimination for isolated FGR (AUC, 0.75 [95% CI, 0.70–0.81]), and provided higher net clinical benefit among 5% to 30% thresholds. In early‐onset PE (n = 14), discrimination was high (AUC, 0.99 [95% CI, 0.98–1.00]); estimates should be interpreted cautiously given the small number of events.

**Conclusion:**

A first‐trimester, mechanistic three‐domain framework captures the pathophysiologic continuum of placental insufficiency and supports accurate, clinically meaningful early risk stratification for PE. Findings were internally validated; external validation—particularly for early‐onset PE—is warranted.

## 1 INTRODUCTION

Pre‐eclampsia (PE) is a complex, multisystemic syndrome and a major cause of maternal and perinatal morbidity worldwide.^1^ Despite decades of research identifying mechanisms such as placental malperfusion, angiogenic imbalance, oxidative stress, and maternal vascular maladaptation, predictive accuracy and therapeutic efficacy remain limited.^2^ Increasing evidence indicates that PE is not a single disease but a spectrum of biologically distinct pathways converging on a common clinical phenotype.^3^

Advances in molecular and systems biology reinforce this concept. High‐dimensional placental omics and single‐cell studies have delineated reproducible subclasses of PE with distinct angiogenic, inflammatory, and immune activation profiles.^4^, ^5^, ^6^ Collectively, these data highlight the biological heterogeneity of PE and the varying contributions of placental and maternal dysfunction.

Translating these discoveries into clinically actionable frameworks remains challenging. Most subclassifications are retrospective and lack integration with noninvasive biomarkers.^7^, ^8^, ^9^, ^10^ Although statistical and machine‐learning models have improved prediction, they remain largely probabilistic and biologically opaque.^11^, ^12^, ^13^, ^14^ Mechanistic frameworks, in contrast, map biomarkers onto physiopathologic domains—angiogenic, uteroplacental, and maternal vascular—enhancing interpretability and translational relevance.^15^, ^16^, ^17^, ^18^ This paradigm shift enables a biologically coherent understanding of PE heterogeneity and may support mechanism‐based prevention.^19^

Translational evidence suggests that placental growth factor (PGF), uterine artery pulsatility index (UtA‐PI), and mean arterial pressure (MAP) capture complementary yet partially independent dimensions of placental and vascular function.^15^, ^16^, ^20^, ^21^ Together, these domains define placental insufficiency and maternal adaptation.

Building on this concept, we aimed to evaluate whether first‐trimester profiles of PGF, UtA‐PI, and MAP, standardized as multiples of the median (MoM), identify complementary domains of dysfunction, and classify PE according to underlying physiopathologic mechanisms, hypothesizing that the cumulative burden of altered domains represents a mechanistic continuum of placental insufficiency associated with progressive disease risk and severity.

## 2 METHODS

### 2.1 Study design and participants

This was a prospective cohort with consecutive recruitment from January 2019 to December 2023 at the Instituto Nacional de Perinatología “Isidro Espinosa de los Reyes” (INPer; national referral center for maternal–fetal medicine) and the Romero Rubio Primary Health Center, Mexico City, Mexico. Singleton pregnancies screened at 11 to 13.6 weeks were included. Eligibility criteria were maternal age ≥18 years, availability of ultrasound, serum and Doppler data, and follow‐up to delivery. Exclusion criteria comprised preexisting chronic kidney disease, autoimmune or cardiovascular disorders, multiple gestations, major fetal structural or chromosomal anomalies, and loss to follow‐up before delivery. The study was approved by the INPer Ethics and Research Committees (Protocol 2021‐1‐38) and conducted in accordance with the Declaration of Helsinki; all participants provided written informed consent. Reporting follows STROBE (Strengthening the Reporting of Observational Studies in Epidemiology) guidelines (Table S1).

### 2.2 Data collection

Maternal demographic, anthropometric, and obstetric data were prospectively recorded in a secure electronic database. Gestational age was determined by fetal crown–rump length at enrollment. Blood pressure was measured in triplicate after 5 minutes of rest using validated automated devices, and MAP was calculated automatically. UtA‐PI was measured transabdominally by certified sonographers, and the mean pulsatility index from both arteries was recorded. Venous blood samples were analyzed for PGF using an automated immunoassay system (Roche Diagnostics GmbH, Cobas e411). Biomarker values (PGF, UtA‐PI, MAP) were log‐transformed and standardized as gestational age–adjusted MoM using regression models derived from women without PE. The algorithm was population‐calibrated to adjust for demographic variability.

### 2.3 Outcomes

The primary outcome was PE, defined according to the American College of Obstetricians and Gynecologists (ACOG) criteria as new‐onset hypertension (systolic ≥140 mm Hg or diastolic ≥90 mm Hg) after 20 weeks of gestation, accompanied by proteinuria (≥300 mg/24 h) or maternal organ dysfunction (renal, hepatic, neurological, or hematologic) or uteroplacental compromise.

PE was subclassified as early‐onset (<34 weeks) or late‐onset (≥34 weeks). Secondary outcomes included fetal growth restriction (FGR), defined as estimated fetal weight or birth weight <10th percentile for gestational age, and a composite outcome (PE or FGR) representing the continuum of placental insufficiency.

### 2.4 Statistical analysis

Analyses were conducted in Stata SE version 18.0 (StataCorp LLC). Continuous variables are reported as median (interquartile range [IQR]) and compared using the Kruskal–Wallis test; categorical variables are shown as frequencies and compared using *χ* ^2^ or Fisher exact test, as appropriate. A two‐tailed *P* value <0.05 was considered statistically significant.

To ensure comparability across gestational age and maternal characteristics, first‐trimester biomarker values were standardized as gestational age–adjusted MoM using regression models derived from the reference (non‐PE) population. Abnormality thresholds were prespecified before outcome inspection at distributional extremes on the MoM scale—low PGF (<p10), high UtA‐PI (>p95), and high MAP (>p95)—in line with first‐trimester screening practice and explicitly not tuned to maximize model fit. Based on these a priori thresholds, we operationalized two complementary mechanistic frameworks. A placental 2 × 2 framework (PGF × UtA‐PI) classified mormo‐placental, angiogenic‐low, uterine resistance, and dual insufficiency phenotypes. Extending this to a three‐domain framework by adding MAP yielded normo (no abnormal domain), molecular (PGF only), hemodynamic (UtA‐PI only), tensional (MAP only), dual (≥2 abnormal domains), and triple (3/3) phenotypes, representing increasing physiopathologic convergence; the three‐domain scheme is nested with respect to the 2 × 2 classification (it collapses to the corresponding placental class when MAP is normal). To verify that domains contributed nonredundant information, we examined pairwise correlations among MoMs and variance inflation factors, and used principal component analysis to visualize independence and variance contribution.

Associations between mechanistic phenotypes and adverse outcomes (PE, FGR, and PE or FGR) were estimated using multivariable logistic regression adjusted for maternal age, body mass index (BMI), parity, smoking, and pregestational diabetes, and expressed as adjusted odds ratios (aORs) with 95% confidence intervals (CIs). Incremental predictive performance was evaluated with nested logistic models that progressively added biomarker domains to a baseline clinical model. Model comparison used Akaike and Bayesian information criteria, likelihood ratio tests, and the area under the (receiver operating characteristic) curve (AUC), with AUC differences assessed by DeLong test. Calibration was examined by observed versus predicted risk among deciles, calibration intercept/slope, and the Brier score. Internal validation employed bootstrap resampling (300 replicates) to derive optimism‐corrected AUCs and 95% CIs. Subtype analyses assessed discrimination for early‐onset (<34 weeks) and late‐onset (≥34 weeks) PE and for isolated FGR (without PE), comparing the baseline clinical model with the integrated three‐domain model. Given the limited number of early‐onset events, subgroup estimates were interpreted with caution. Clinical utility was assessed with decision curve analysis, quantifying net benefit for the clinical model (A) and the integrated model (D) among clinically relevant thresholds (5%–30%), using “treat all” and “treat none” as references.

## 3 RESULTS

### 3.1 Study population and baseline characteristics

A total of 1925 pregnancies were included, and 104 (5.4%) developed PE. Among these, 14 (13.5%) were early‐onset (<34 weeks) and 90 (86.5%) were late‐onset (≥34 weeks). Additionally, 56 pregnancies (3.1%) presented with isolated FGR in the absence of PE.

Gestational age at sampling was similar but affected women were older, had higher BMI, and delivered earlier. In the PE group, MAP and UtA‐PI were higher, while PGF levels were lower. After gestational age standardization, median MoM values differed significantly: PGF 0.71 (IQR, 0.52–1.05) versus 1.02 (IQR, 0.77–1.31); UtA‐PI 1.08 (IQR, 0.85–1.34) versus 1.01 (IQR, 0.80–1.26); and MAP 1.09 (IQR, 1.03–1.15) versus 1.00 (IQR, 0.94–1.07) (all *P* < 0.01). Chronic hypertension, antiphospholipid syndrome, and prior PE were also more frequent among affected women (Table 1).

**TABLE 1:** Maternal characteristics and biomarkers according to PE status.

Characteristic	No PE (*n* = 1821)	PE (*n* = 104)	*P* value
Age (years)	28.65 (22.30–34.25)	32.32 (25.28–36.88)	<0.001
BMI (kg/m^2^)	26.02 (23.05–29.45)	29.13 (26.02–32.73)	<0.001
Gestational age at sampling (weeks)	12.74 (12.25–13.20)	12.85 (12.12–13.35)	0.230
Gestational age at delivery (weeks)	38.55 (37.57–39.45)	36.60 (35.60–37.85)	<0.001
UtA‐PI	1.48 (1.19–1.87)	1.62 (1.22–2.02)	0.008
MAP (mm Hg)	75.00 (69.42–80.58)	84.08 (79.99–86.67)	<0.001
PGF (pg/mL)	23.40 (17.23–32.72)	18.18 (11.73–22.67)	<0.001
UtA MoM	1.01 (0.80–1.26)	1.08 (0.85–1.34)	0.005
MAP MoM	1.00 (0.94–1.07)	1.09 (1.03–1.15)	<0.001
PGF MoM	1.02 (0.77–1.31)	0.71 (0.52–1.05)	<0.001
Chronic hypertension	61 (3.35%)	11 (10.58%)	<0.001
Antiphospholipid syndrome	5 (0.27%)	4 (3.85%)	0.001
Previous PE	97 (5.33%)	21 (20.19%)	<0.001
Hypothyroidism	197 (10.82%)	2 (1.92%)	0.003
Pregestational diabetes	99 (5.44%)	8 (7.69%)	0.358
Primigravida	604 (33.17%)	36 (34.61%)	0.872
Tobacco use	92 (5.05%)	6 (5.77%)	0.784
Alcohol use	23 (1.26%)	1 (0.96%)	0.772
Drug use	24 (1.32%)	2 (1.92%)	0.623
Lupus	11 (0.60%)	0 (0.00%)	0.422
Polycystic ovary syndrome	37 (2.03%)	3 (2.88%)	0.564
Heart disease	31 (1.70%)	0 (0.00%)	0.175
IVF/ICSI conception	13 (0.71%)	0 (0.00%)	0.382
Ovulation induction	17 (0.93%)	0 (0.00%)	0.317

*Note*: Values are expressed as median (interquartile range) or *n* (%). *P* values were obtained with the Kruskal–Wallis test (continuous) or *χ* ^2^/Fisher exact test (categorical).

Abbreviations: BMI, body mass index; MAP, mean arterial pressure; MoM, multiples of the median; PE, pre‐eclampsia; PGF, placental growth factor; ICSI, intracytoplasmic sperm injection; IVF, in vitro fertilization; UtA, uterine artery; UtA‐PI, uterine artery pulsatility index.

**FIGURE 1:**
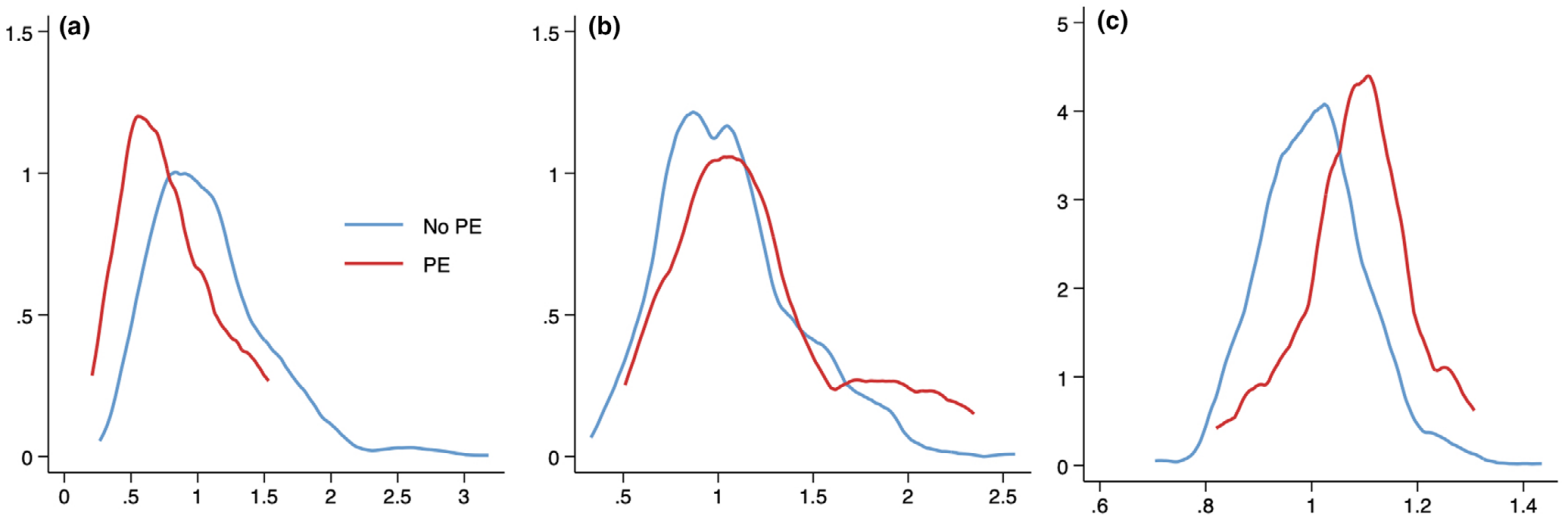
Kernel density distributions of PGR MoM (a), UtA‐PI MoM (b), and MAP MoM (c) by PE status. MAP, mean arterial pressure; MoM, multiples of the median; PE, pre‐eclampsia; PGF, placental growth factor; UtA‐PI, uterine artery pulsatility index.

Kernel density plots revealed a left shift of PGF MoM and right shifts of UtA‐PI and MAP MoMs in PE (Figure 1), consistent with early molecular, uteroplacental, and maternal‐tensional dysfunction. Pairwise correlations were weak (|r| ≤ 0.13), and variance inflation factors were ≈1.0, confirming statistical independence among domains (Figure S1, Table S2). Principal component analysis identified three orthogonal components explaining 38.9%, 32.2%, and 28.9% of the variance, corresponding respectively to the uteroplacental, maternal vascular, and molecular/angiogenic axes (Figure S2).

### 3.2 Physiopathologic phenotypes of placental dysfunction

Pregnancies were classified into mechanistic phenotypes based on percentile thresholds (PGF <p10, UtA‐PI >p95, MAP >p95). In the 2 × 2 framework (PGF + UtA‐PI), 86.8% were normo‐placental, 8.2% nngiogenic‐low, 3.4% uterine‐resistance, and 1.7% dual insufficiency; the prevalence of PE increased stepwise across these groups from 4.2% to 34.4% (Table S3).

**FIGURE 2:**
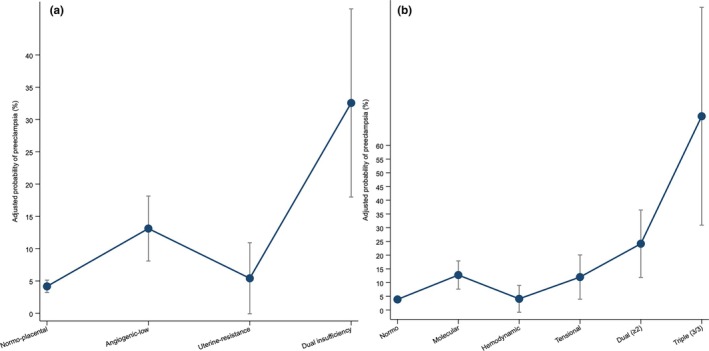
Adjusted pre‐eclampsia risk by phenotype: 2 × 2 (a) and three‐domain (b); points = probabilities (95% confidence intervals).

In the three‐domain model, phenotype distribution was normo (81.7%), molecular (7.6%), hemodynamic (3.2%), tensional (5.1%), dual (≥2 altered domains, 2.1%), and triple (3/3, 0.3%). The risk of PE increased progressively from 3.9% (normo) to 12.9% (molecular), 9.2% (tensional), 24.4% (dual), and 80.0% (triple) (*P* for trend <0.001) (Table 2). After adjustment for maternal factors, the molecular and dual/triple phenotypes showed the highest odds of PE. Figure 2 displays adjusted probabilities (95% CI), confirming a stepwise risk gradient.

**TABLE 2:** PE and placental insufficiency outcomes according to three‐domain phenotypes (PGF MoM p10/UtA‐PI MoM p95/MAP MoM p95).

Phenotype (3 domains)	*n* (%)	PE, *n*/*N* (%)	FGR, *n*/*N* (%)	SGA, *n*/*N* (%)	PE or FGR, *n*/*N* (%)
Normo	1572 (81.7)	61/1572 (3.88)	47/1572 (2.99)	154/1572 (9.80)	105/1572 (6.68)
Molecular (PGF MoM <p10)	147 (7.6)	19/147 (12.93)	11/147 (7.48)	28/147 (19.05)	29/147 (19.73)
Hemodynamic (UtA MoM >p95)	62 (3.2)	3/62 (4.84)	1/62 (1.61)	8/62 (12.90)	4/62 (6.45)
Tensional (MAP MoM >p95)	98 (5.1)	9/98 (9.18)	3/98 (3.06)	10/98 (10.20)	8/98 (8.16)
Dual (≥2 altered domains)	41 (2.1)	10/41 (24.39)	0/41 (0.00)	6/41 (14.63)	10/41 (24.39)
Triple (3/3 altered)	5 (0.3)	4/5 (80.00)	2/5 (40.00)	2/5 (40.00)	4/5 (80.00)
Total	1925 (100)	106/1925 (5.51)	64/1925 (3.32)	208/1925 (10.81)	164/1925 (8.52)

*Note*: Phenotype definitions: normo, all domains normal (PGF MoM ≥p10, UtA‐PI MoM ≤ p95, MAP MoM ≤ p95); molecular, isolated angiogenic deficiency (PGF MoM <p10 only); hemodynamic, isolated uteroplacental resistance (UtA‐PI MoM >p95 only); tensional, isolated maternal vascular load (MAP MoM >p95 only); dual (≥2), coexistence of two abnormal domains; triple (3/3), simultaneous alteration of all domains. Values are expressed as *n*/*N* (%). Phenotypes were derived from gestational age–adjusted MoM for PGF, UtA‐PI, and MAP. The composite outcome PE ∨ FGR represents PE or FGR.

Abbreviations: FGR, fetal growth restriction; MAP, mean arterial pressure; MoM, multiples of the median; PE, pre‐eclampsia; PGF, placental growth factor; SGA, small for gestational age; UtA, uterine artery; UtA‐PI, uterine artery pulsatility index.

Modeling the cumulative number of altered domains (0–3) revealed an exponential increase in PE probability—from 3.8% with none to 53.0% when all three domains were abnormal—illustrating a continuous, quantifiable spectrum of placental dysfunction (Figure S3).

### 3.3 Predictive performance of mechanistic models

Nested logistic models assessed the incremental predictive value of domain integration (Table 3). The baseline clinical model (A) achieved an AUC of 0.68; adding UtA‐PI (B) increased the AUC to 0.70, adding PGF (C) increased the AUC to to 0.75, and integrating all three domains (D) increased the AUC to 0.81 (95% CI 0.77–0.86; *P* < 0.001 vs. model A).

**TABLE 3:** Incremental performance of nested models for PE.

Model	Predictors	No.	df	Log‐likelihood (model)	AIC	BIC	AUC	LR test (vs previous model)	ROC vs. A	Calibration (D only)	Bootstrap (D)
A	Clinical: maternal age, BMI, primigravida, smoking, pregestational diabetes	1920	6	−390.579	793.158	826.518	0.677	n/a (baseline)	n/a (baseline)	n/a	n/a
B	A + UtA MoM	1920	7	−383.859	781.717	820.634	0.702	n/a (different N)	n/a	n/a	n/a
C	A + PGF MoM	1920	7	−357.334	728.669	767.523	0.745	n/a (different N)	n/a	n/a	n/a
D	A + PGF MoM + UtA MoM + MAP MoM	1920	9	−328.375	674.750	724.706	0.812	C → D: LR‐*χ* ^2^(2) = 57.92, *P* < 0.001	A vs. D: *P* < 0.001	HL *χ* ^2^(8) = 20.56, *P* = 0.0084; intercept≈0; slope≈1.00	AUC 0.812 ± 0.023 (95% CI 0.767–0.856, 298 reps)

*Note*: Model A included clinical variables only (maternal age, BMI, parity, smoking, pregestational diabetes). Models B–D sequentially added UtA‐PI MoM, PGF MoM, and MAP MoM. AUC values were obtained from the roctab procedure: A = 0.677, B = 0.702, C = 0.745, D = 0.812. The likelihood ratio test was significant for model C → D (LR‐*χ* ^2^ = 57.9, *P* < 0.001); comparisons A → B and A → C were not performed because sample sizes differed. ROC analysis confirmed superior discrimination for models D vs. A (*P* < 0.001). Model D was well‐calibrated (intercept≈0; slope≈1; HL *χ* ^2^(8) = 20.6, *P* = 0.008) and showed stable discrimination after bootstrap validation (298 replicates; mean AUC 0.812 ± 0.023 [95% CI, 0.767–0.856]). All predictive models were fitted on the subset of 1920 pregnancies with complete data for all covariates and biomarkers (five observations with missing PGF, UtA‐PI, MAP, or clinical variables were excluded).

Abbreviations: AIC, Akaike information criterion; AUC, area under the (receiver operating characteristic) curve; BIC, Bayesian information criterion; BMI, body mass index; HL, Hosmer–Lemeshow; LR, likelihood ratio; MAP, mean arterial pressure; MoM, multiples of the median; n/a, not available; PE, pre‐eclampsia; PGF, placental growth factor; UtA, uterine artery.

**FIGURE 3:**
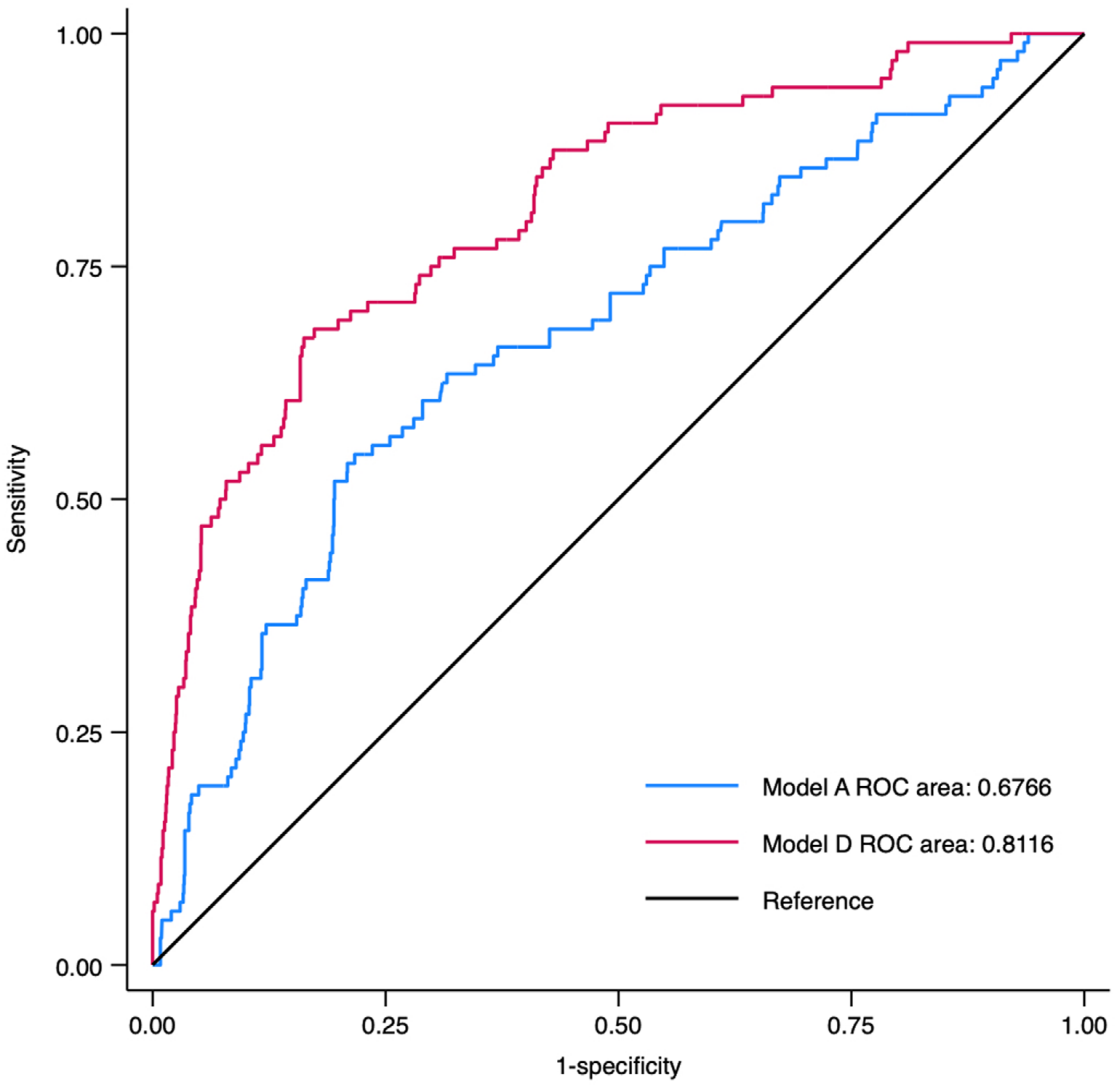
ROC curves for models A–D (A = clinical; D = clinical + PGF MoM + UtA‐PI MoM + MAP MoM). MAP, mean arterial pressure; MoM, multiples of the median; PGF, placental growth factor; ROC, receiver operating characteristic [curve]; UtA‐PI, uterine artery pulsatility index.

Model D demonstrated excellent calibration (intercept −0.005 [95% CI, −0.025 to 0.016]; slope 1.08 [95% CI, 0.85–1.32]) and a Brier score of 0.045, indicating close agreement between predicted and observed risks (Table S4, Figure S4). Bootstrap validation (298 replicates) confirmed model stability (AUC, 0.812 ± 0.023 [95% CI, 0.767–0.856]) (Figure 3).

### 3.4 Subtype and secondary outcomes

The integrated model achieved near‐perfect discrimination for early‐onset PE (AUC, 0.99 [95% CI, 0.98–1.00]) and moderate discrimination for late‐onset disease (AUC, 0.78 [95% CI, 0.73–0.83]). Among pregnancies without PE, it predicted isolated FGR with good accuracy (AUC, 0.75 [95% CI, 0.70–0.81]), outperforming the clinical model (AUC, 0.67; *P* < 0.01) (Table 4; Figure S5). The probability of isolated FGR increased with the number of altered domains, from 2.9% (0 domains) to 3.5% (three domains, supporting a continuous physiopathologic gradient; Table S5).

**TABLE 4:** Discrimination of the integrated three‐domain model (model D) versus the clinical model (model A) for ePE, lPE, and iFGR.

Outcome	Events (*n*)	Model A AUC (95% CI)	Model D AUC (95% CI)	ΔAUC	*P* value (DeLong)
ePE	14	0.86 (0.74–0.98)	0.99 (0.98–1.00)	+0.13	<0.001
lPE	90	0.66 (0.59–0.74)	0.78 (0.73–0.83)	+0.12	<0.001
iFGR	56	0.67 (0.60–0.74)	0.75 (0.70–0.81)	+0.08	0.006

*Note*: Model A (clinical) = maternal age, body mass index, parity, smoking, pregestational diabetes; model D (integrated) = model A + PGF MoM + UtA‐PI MoM + MAP MoM. AUCs from ROC analysis; *P* values from DeLong test comparing model D vs. model A; ΔAUC = (model D − model A).

Abbreviations: AUC, area under the (receiver operating characteristic) curve; ePE, early‐onset pre‐eclampsia (<34 weeks); FGR, fetal growth restriction; iFGR, isolated fetal growth restriction; FGR (no pre‐eclampsia); lPE, late‐onset pre‐eclampsia (≥34 weeks); PE, pre‐eclampsia; PGF, placental growth factor; MoM, multiples of the median; ROC, receiver operating characteristic [curve]; UtA‐PI, uterine artery pulsatility index.

### 3.5 Clinical utility

**FIGURE 4:**
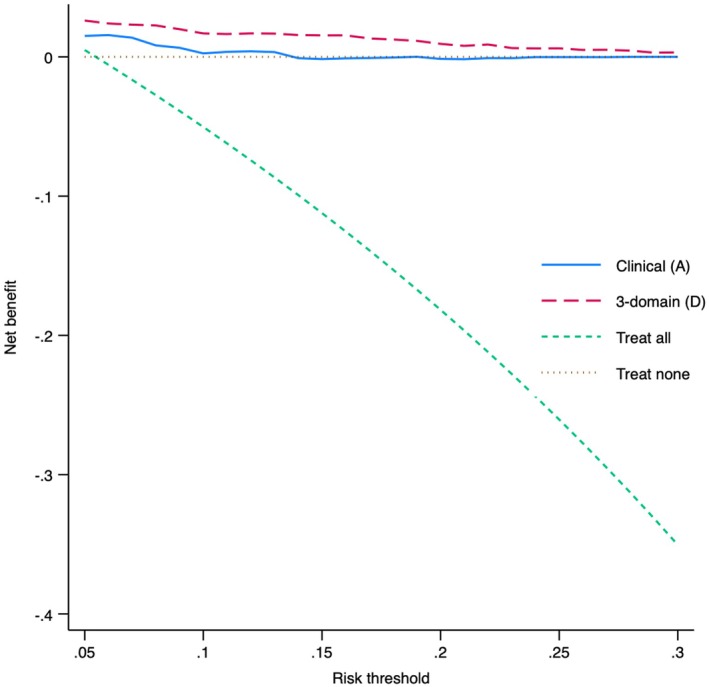
Decision curve analysis (thresholds 5%–30%): Net benefit of clinical‐only model (A) vs. three‐domain model (D), with treat all and treat none as references; D shows higher net benefit across the range.

Decision curve analysis showed that the integrated three‐domain model provided higher net clinical benefit than the clinical‐only model among decision thresholds from 5% to 30% and consistently outperformed both treat‐all and treat‐none strategies (Figure 4, Table S6), confirming its potential for integration into early clinical screening.

## 4 DISCUSSION

### 4.1 Main findings

We present a mechanistic first‐trimester framework that integrates angiogenic, uteroplacental, and maternal vascular domains to position PE on a measurable continuum of placental dysfunction. Risk increased stepwise with the number of altered domains, from isolated molecular impairment to global placental–maternal dysfunction. The integrated model showed excellent overall discrimination (AUC, ≈0.81) and correctly stratified early‐onset PE, consistent with its stronger placental origin, whereas performance for late‐onset disease was moderate, reflecting greater maternal vascular involvement. The same framework also identified isolated FGR, supporting a shared physiopathologic continuum that extends beyond clinically manifest PE.

### 4.2 Comparison with existing literature

Our results align with the shift from early/late‐onset labels to mechanistic, domain‐based stratification. Precision‐screening approaches that combine maternal factors with MAP, uterine artery Doppler, and PGF outperform single‐parameter models and underpin the FMF “triple test” concept.^19^, ^22^, ^23^ Midgestation data complement this paradigm: high UtA‐PI remains a strong signal for early‐onset PE even under aspirin and interacts with comorbidities,^24^ and reassessment of Doppler refines risk after first‐trimester screening, although it cannot capture the full spectrum alone.^25^ At the molecular level, longitudinal proteomics map angiogenic, inflammatory, and metabolic pathways to specific trajectories,^7^ and convergent evidence supports angiogenic imbalance, defective spiral artery remodeling, and systemic endothelial activation as interdependent mechanisms.^9^ These signals are consistent with our three‐axis model and here are translated into measurable in vivo biomarkers. Because biomarker distributions are population‐dependent, MoM calibration is critical. Our previous Latin American equations for PGF, UtA‐PI, and MAP provided the standardization used in this analysis.^26^ Additional studies reinforce the interpretability of each domain: serial PGF with Doppler improves diagnostic accuracy and delineates perfusion patterns^15^; late‐pregnancy vascular indices reflect persistent cardiovascular maladaptation^17^; a maternal cardiovascular‐origin hypothesis reframes the placenta as “victim” when maternal performance is suboptimal^18^; and retinal microvascular metrics plus MAP capture systemic endothelial dysfunction early in pregnancy.^27^ These observations are concordant with our prior machine‐learning work in a Latin American cohort integrating maternal characteristics with locally calibrated MoMs for PGF, UtA‐PI, and MAP (preterm‐PE AUC, 0.90; early‐onset PE AUC, 0.96).^13^ Altogether, available evidence and our data support the biological complementarity of angiogenic, uteroplacental, and maternal vascular domains.

### 4.3 Clinical translation and risk communication

This framework is intended to trigger proportionate, evidence‐based care, not merely to label risk. In individuals meeting high‐risk criteria, low‐dose aspirin remains the preventive action with the strongest evidence for preterm PE and should be started after 12 weeks—ideally before 16 weeks; our first‐trimester approach operationalizes timely identification for that window.^22^, ^23^ Beyond prophylaxis, risk‐informed pathways support blood pressure optimization and closer fetal growth/Doppler surveillance with predefined escalation and referral thresholds. Because risk disclosure can cause anxiety, we use a structured strategy: communicate absolute (not relative) risk and its uncertainty, explain that screening reflects mechanistic signals rather than a diagnosis, discuss false‐positive/false‐negative trade‐offs and the concrete next steps, and apply shared decision‐making with access to psychosocial support. In our cohort, decision curve analysis (Figure 4) demonstrated higher net clinical benefit of the three‐domain model than treat‐all/treat‐none and clinical‐only approaches among 5% to 30% thresholds; accordingly, we restrict the “high‐risk” designation to ranges where net benefit is positive, supporting useful and proportionate clinical action.

### 4.4 Implementation in resourcelimited settings

Implementation in low‐ and middle‐income settings is feasible with a MAP‐first strategy that leverages routine first‐trimester care at minimal cost. MAP obtained at 11 to 13 + 6 weeks can be standardized to gestational age–adjusted MoMs using locally derived reference equations, minimizing population‐related misclassification.^26^ This minimal configuration—maternal factors plus MAP—provides an immediate entry point for risk stratification without additional laboratory infrastructure and is consistent with first‐trimester screening practice.^21^, ^22^ As capacity matures, uterine artery pulsatility index can be incorporated with basic quality assurance and expressed as MoM to create a dual‐domain screen; where feasible, PGF is subsequently added to complete the three‐domain model. Programs can conserve resources through reflex testing (MAP‐ or dual domain–based triage to PGF), batched assays with predefined turnaround times, and offline or electronic medical record–embedded calculators that apply locally calibrated MoM thresholds uniformly across tiers of care. Short pilot phases to verify calibration and workflow, coupled with periodic monitoring of MoM distributions, mitigate drift and support scale‐up. This staged plan preserves the mechanistic interpretability of the framework while matching resources to local capacity, offering a realistic and equitable route to adoption in resource‐constrained health systems.

### 4.5 Strengths and limitations of the study

This prospective cohort with standardized first‐trimester biomarker assessment provides robust evidence linking angiogenic, uteroplacental, and maternal vascular domains to early placental dysfunction. Locally calibrated MoMs minimized demographic bias, and bootstrap internal validation confirmed model stability and calibration. Decision curve analysis demonstrated superior clinical utility of the integrated three‐domain model compared with a clinical‐only approach, underscoring its potential for early risk stratification. Key limitations include the single‐country setting, which may restrict external generalizability of percentile thresholds, and the limited availability of PGF testing in routine care. External, multiethnic validation and implementation studies are required to confirm reproducibility and feasibility among healthcare systems. Finally, the early‐onset PE subgroup was small (n = 14), which may inflate discrimination estimates and widens uncertainty. Although internal bootstrap validation supports stability, these early‐onset results should be interpreted cautiously and confirmed in larger, external cohorts before broad implementation.

## 5 CONCLUSION

This study introduces a mechanistic first‐trimester framework conceptualizing PE as a continuum of angiogenic, uteroplacental, and maternal vascular dysfunction. Quantifying these domains as MoMs captures early placental insufficiency and enables mechanistic risk stratification for precision obstetrics.

## AUTHOR CONTRIBUTIONS

Johnatan Torres‐Torres conceived the study, designed the mechanistic framework, and performed the formal analysis. Salvador Espino‐y‐Sosa and Raigam Jafet Martinez‐Portilla contributed to study conceptualization, bioinformatics design, and critical manuscript revision. Elsa Romelia Moreno‐Verduzco, Irma Eloisa Monroy‐Muñoz, Juan Mario Solis‐Paredes, and Javier Pérez Duran collected and validated the clinical data. Héctor Borboa‐Olivares and Lourdes Rojas‐Zepeda oversaw clinical implementation and data interpretation. All authors meet the ICMJE criteria for authorship, contributed intellectually to the work, critically reviewed the manuscript, and approved the final version for submission.

## FUNDING INFORMATION

The marker databases analyzed in this study were financed with the Public Health Services of Mexico City budget allocated to the first‐trimester pregnancy screening program (POA 2019). This funding body had no involvement in the study design; in the collection, analysis, or interpretation of data; in the writing of the manuscript; or in the decision to submit the article for publication.

## CONFLICT OF INTEREST STATEMENT

The authors declare that they have no conflicts of interest related to this work.

## ETHICS STATEMENT

This study was approved by the institutional ethics and research committees of the *Instituto Nacional de Perinatología “Isidro Espinosa de los Reyes”* (Protocol No. 2021‐1‐38). All procedures were conducted in accordance with the principles of the Declaration of Helsinki and its later amendments. Written informed consent was obtained from all participants before enrollment.

## PROTOCOL AND REGISTRATION

The study followed the approved institutional research protocol (INPer No. 2021‐1‐38). It forms part of the longitudinal program for first‐trimester screening and prediction of placental disorders developed at the *Instituto Nacional de Perinatología “Isidro Espinosa de los Reyes”*.

## GUARANTOR

Johnatan Torres‐Torres serves as the guarantor of this work and accepts full responsibility for the integrity of the data and the accuracy of the analyses.

## Supporting information


Data S1:

## Data Availability

The data sets generated and analyzed during the present study are available from the corresponding author upon reasonable request. Data access will be granted to qualified researchers for legitimate scientific use. Requests should be addressed to torresmmf@gmail.com.
