# Supplementary material for: A first‐trimester mechanistic framework integrating three Physiopathologic biomarker domains for pre‐eclampsia classification
Source: Int J Gynaecol Obstet. 2026 Jan 19;174(1):182–91. doi: 10.1002/ijgo.70804 (PMC13278659; doi:10.1002/ijgo.70804)
Supplement: Supplementary file 1 — Data S1: [file IJGO-174-182-s001.docx]

| Table S1. STROBE Statement—Checklist of items that should be included in reports of *cohort studies* | | | |
| --- | --- | --- | --- |
|  | Item No | Recommendation | Page(s) in manuscript |
| Title and abstract | 1 | (*a*) Indicate the study’s design with a commonly used term in the title or the abstract | 1 |
|  |  | (*b*) Provide in the abstract an informative and balanced summary of what was done and what was found | 1-2 |
| Introduction | | |  |
| Background/rationale | 2 | Explain the scientific background and rationale for the investigation being reported | 3-4 |
| Objectives | 3 | State specific objectives, including any prespecified hypotheses | 4 |
| Methods | | |  |
| Study design | 4 | Present key elements of study design early in the paper | 5 |
| Setting | 5 | Describe the setting, locations, and relevant dates, including periods of recruitment, exposure, follow-up, and data collection | 5-6 |
| Participants | 6 | (*a*) Give the eligibility criteria, and the sources and methods of selection of participants. Describe methods of follow-up | 5-6 |
|  |  | (*b*) For matched studies, give matching criteria and number of exposed and unexposed | (not applicable) |
| Variables | 7 | Clearly define all outcomes, exposures, predictors, potential confounders, and effect modifiers. Give diagnostic criteria, if applicable | 6-7 |
| Data sources/ measurement | 8* | For each variable of interest, give sources of data and details of methods of assessment (measurement). Describe comparability of assessment methods if there is more than one group | *6-7* |
| Bias | 9 | Describe any efforts to address potential sources of bias | 7-8 |
| Study size | 10 | Explain how the study size was arrived at | 8 |
| Quantitative variables | 11 | Explain how quantitative variables were handled in the analyses. If applicable, describe which groupings were chosen and why | 8 |
| Statistical methods | 12 | (*a*) Describe all statistical methods, including those used to control for confounding | 8-10 |
|  |  | (*b*) Describe any methods used to examine subgroups and interactions | 9-10 |
|  |  | (*c*) Explain how missing data were addressed | 9-10 |
|  |  | (*d*) If applicable, explain how loss to follow-up was addressed | 5, 9 |
|  |  | (*e*) Describe any sensitivity analyses | 9-10 |
| Results | | |  |
| Participants | 13* | (a) Report numbers of individuals at each stage of study—eg numbers potentially eligible, examined for eligibility, confirmed eligible, included in the study, completing follow-up, and analysed | 10-11 |
|  |  | (b) Give reasons for non-participation at each stage | 10-11 |
|  |  | (c) Consider use of a flow diagram | 10-11 |
| Descriptive data | 14* | (a) Give characteristics of study participants (eg demographic, clinical, social) and information on exposures and potential confounders | 11-12 |
|  |  | (b) Indicate number of participants with missing data for each variable of interest | 11-12 |
|  |  | (c) Summarise follow-up time (eg, average and total amount) | 11-12 |
| Outcome data | 15* | Report numbers of outcome events or summary measures over time | 12-14 |
| Main results | 16 | (*a*) Give unadjusted estimates and, if applicable, confounder-adjusted estimates and their precision (eg, 95% confidence interval). Make clear which confounders were adjusted for and why they were included | 12-14 |
|  |  | (*b*) Report category boundaries when continuous variables were categorized | 12-13 |
|  |  | (*c*) If relevant, consider translating estimates of relative risk into absolute risk for a meaningful time period | 12-13 |
| Other analyses | 17 | Report other analyses done—eg analyses of subgroups and interactions, and sensitivity analyses | 14-15 |
| Discussion | | |  |
| Key results | 18 | Summarise key results with reference to study objectives | 15-16 |
| Limitations | 19 | Discuss limitations of the study, taking into account sources of potential bias or imprecision. Discuss both direction and magnitude of any potential bias | 17-18 |
| Interpretation | 20 | Give a cautious overall interpretation of results considering objectives, limitations, multiplicity of analyses, results from similar studies, and other relevant evidence | 18-19 |
| Generalisability | 21 | Discuss the generalisability (external validity) of the study results | 19 |
| Other information | | |  |
| Funding | 22 | Give the source of funding and the role of the funders for the present study and, if applicable, for the original study on which the present article is based | 21-22 |
| *Give information separately for exposed and unexposed groups.  Note: An Explanation and Elaboration article discusses each checklist item and gives methodological background and published examples of transparent reporting. The STROBE checklist is best used in conjunction with this article (freely available on the Web sites of PLoS Medicine at http://www.plosmedicine.org/, Annals of Internal Medicine at http://www.annals.org/, and Epidemiology at http://www.epidem.com/). Information on the STROBE Initiative is available at http://www.strobe-statement.org. | | | |

| **Table S2.** Multicollinearity diagnostics among biomarker domains (PlGF MoM, UtA-PI MoM, MAP MoM) based on a linear auxiliary model with centered variance-inflation factors (VIF). | | | | | | |
| --- | --- | --- | --- | --- | --- | --- |
| Predictor | Coefficient (β) | SE | t | p-value | VIF | 1/VIF |
| PlGF MoM | −0.069 | 0.0116 | −5.94 | <0.001 | 1.02 | 0.98 |
| UtA-PI MoM | +0.042 | 0.0140 | 3.01 | 0.003 | 1.02 | 0.98 |
| MAP MoM | +0.364 | 0.0497 | 7.31 | <0.001 | 1.00 | 0.99 |
| *Constant* | −0.284 | 0.054 | −5.21 | <0.001 | — | — |
| Model summary: F(3, 1898)=36.9; p<0.001; R² = 0.055; Adj R² = 0.054; n = 1,925 observations.  All centered VIFs ≈ 1.0 indicated negligible collinearity, confirming statistical independence among the three biomarker domains. | | | | | | |

| **Table S3.** Preeclampsia and placental insufficiency outcomes according to 2×2 phenotypes (PLGFmom p10 / UTAmom p95) | | | | | |
| --- | --- | --- | --- | --- | --- |
| Phenotype (2×2) | n (%) | PEn/N (%) | FGRn/N (%) | SGAn/N (%) | PE ∨ FGRn/N (%) |
| Normo-placental | 1,670 (86.8) | 70 / 1,670 (4.19) | 50 / 1,670 (2.99) | 164 / 1,670 (9.82) | 117 / 1,670 (7.01) |
| Angiogenic-low (PLGF mom <p10) | 158 (8.2) | 21 / 158 (13.29) | 11 / 158 (6.96) | 29 / 158 (18.35) | 31 / 158 (19.62) |
| Uterine-resistance (UTA mom >p95) | 65 (3.4) | 4 / 65 (6.15) | 1 / 65 (1.54) | 8 / 65 (12.31) | 5 / 65 (7.69) |
| Dual insufficiency (PLGFmom<p10 & UTAmom>p95) | 32 (1.7) | 11 / 32 (34.38) | 2 / 32 (6.25) | 7 / 32 (21.88) | 11 / 32 (34.38) |
| Total | 1,925 (100) | 106 / 1,925 (5.51) | 64 / 1,925 (3.32) | 208 / 1,925 (10.81) | 164 / 1,925 (8.52) |
| PE = preeclampsia; FGR = fetal growth restriction; SGA = small for gestational age.  N*ormo-placental*: PlGFmom ≥p10 and UTAmom ≤p95.  *Angiogenic-low*: PlGFmom <p10 and UTAmom ≤p95.  *Uterine-resistance*: PlGFmom ≥p10 and UTAmom >p95.  *Dual insufficiency*: PlGFmom <p10 and UTAmom >p95. | | | | | |

| **Table S4.** Calibration model D | | | | | | |
| --- | --- | --- | --- | --- | --- | --- |
| decD | Mean pred | events | n | Obs rate | lo | hi |
| 1 | 0.002512512273334 | 1 | 191 | 0.0052356021 | 0.0009248109739091 | 0.02905576367034 |
| 2 | 0.0064386560293471 | 4 | 190 | 0.021052632 | 0.0082166836621129 | 0.0528716887240759 |
| 3 | 0.011223160088509 | 1 | 190 | 0.0052631581 | 0.0009296816231366 | 0.0292055587980307 |
| 4 | 0.0158018651078089 | 2 | 190 | 0.010526316 | 0.0028914581610846 | 0.0375614929150899 |
| 5 | 0.0223615389622332 | 4 | 190 | 0.021052632 | 0.0082166836621129 | 0.0528716887240759 |
| 6 | 0.0305680379343987 | 11 | 191 | 0.057591625 | 0.0324591758672226 | 0.1001689599043580 |
| 7 | 0.044528924519075 | 7 | 190 | 0.036842104 | 0.0179588912226093 | 0.0740826076514046 |
| 8 | 0.0671481567108589 | 4 | 190 | 0.021052632 | 0.0082166836621129 | 0.0528716887240759 |
| 9 | 0.1049102822178260 | 18 | 190 | 0.094736844 | 0.0607671049583043 | 0.1447692129465200 |
| 10 | 0.2417011790540100 | 52 | 190 | 0.2736842 | 0.2152449281406510 | 0.3410935191712390 |

| **Table S5.** Adjusted probability of isolated fetal growth restriction (iFGR) according to the cumulative number of altered physiopathologic domains | | | | |
| --- | --- | --- | --- | --- |
| Number of altered domains | n (%) in cohort (n = 1,821) | Adjusted probability of iFGR (%) | 95 % CI | p value |
| 0 | 1,572 (86.3) | 2.91 | 2.11 – 3.71 | Reference |
| 1 | 284 (15.6) | 3.09 | 1.73 – 4.45 | 0.41 |
| 2 | 41 ( 2.2 ) | 3.29 | 0.50 – 6.08 | 0.21 |
| 3 | 5 ( 0.3 ) | 3.50 | –0.99 – 7.98 | 0.13 |
| iFGR, isolated fetal growth restriction; n_dom, number of altered physiopathologic domains (low PlGF MoM <p10, high UtA-PI MoM >p95, high MAP MoM >p95). Logistic regression adjusted for maternal age, BMI, parity, smoking, and pregestational diabetes. Predicted probabilities obtained from marginal effects analysis; 95 % CIs by delta method. p values refer to comparison with the reference category. | | | | |

| **Table S6.** Net benefit values according to decision thresholds for the clinical (A) and integrated three-domain (D) models | | | | |
| --- | --- | --- | --- | --- |
| Decision threshold | NB — Clinical model (A) | NB — 3-domain model (D) | Treat-all strategy | Treat-none strategy |
| 0.05 | 0.0150 | 0.0261 | 0.0049 | 0 |
| 0.06 | 0.0156 | 0.0240 | –0.0057 | 0 |
| 0.07 | 0.0137 | 0.0231 | –0.0165 | 0 |
| 0.08 | 0.0082 | 0.0226 | –0.0275 | 0 |
| 0.09 | 0.0065 | 0.0199 | –0.0388 | 0 |
| 0.10 | 0.0025 | 0.0168 | –0.0504 | 0 |
| 0.11 | 0.0036 | 0.0164 | –0.0622 | 0 |
| 0.12 | 0.0040 | 0.0168 | –0.0742 | 0 |
| 0.13 | 0.0034 | 0.0167 | –0.0866 | 0 |
| 0.14 | –0.0009 | 0.0157 | –0.0992 | 0 |
| 0.15 | –0.0016 | 0.0154 | –0.1121 | 0 |
| 0.16 | –0.0011 | 0.0154 | –0.1254 | 0 |
| 0.17 | –0.0008 | 0.0132 | –0.1389 | 0 |
| 0.18 | –0.0005 | 0.0125 | –0.1528 | 0 |
| 0.19 | 0.0000 | 0.0115 | –0.1671 | 0 |
| 0.20 | –0.0014 | 0.0093 | –0.1817 | 0 |
| 0.21 | –0.0017 | 0.0079 | –0.1966 | 0 |
| 0.22 | –0.0009 | 0.0089 | –0.2119 | 0 |
| 0.23 | –0.0009 | 0.0063 | –0.2277 | 0 |
| 0.24 | –0.0002 | 0.0061 | –0.2438 | 0 |
| 0.25 | –0.0002 | 0.0061 | –0.2604 | 0 |
| 0.26 | –0.0002 | 0.0050 | –0.2775 | 0 |
| 0.27 | –0.0002 | 0.0051 | –0.2950 | 0 |
| 0.28 | 0.0000 | 0.0045 | –0.3129 | 0 |
| 0.29 | 0.0000 | 0.0030 | –0.3314 | 0 |
| 0.30 | 0.0000 | 0.0032 | –0.3505 | 0 |
| NB = net benefit. The “treat-none” strategy yields NB = 0 across all thresholds, serving as the reference line. Positive values indicate a net clinical advantage over treating none. | | | | |

**
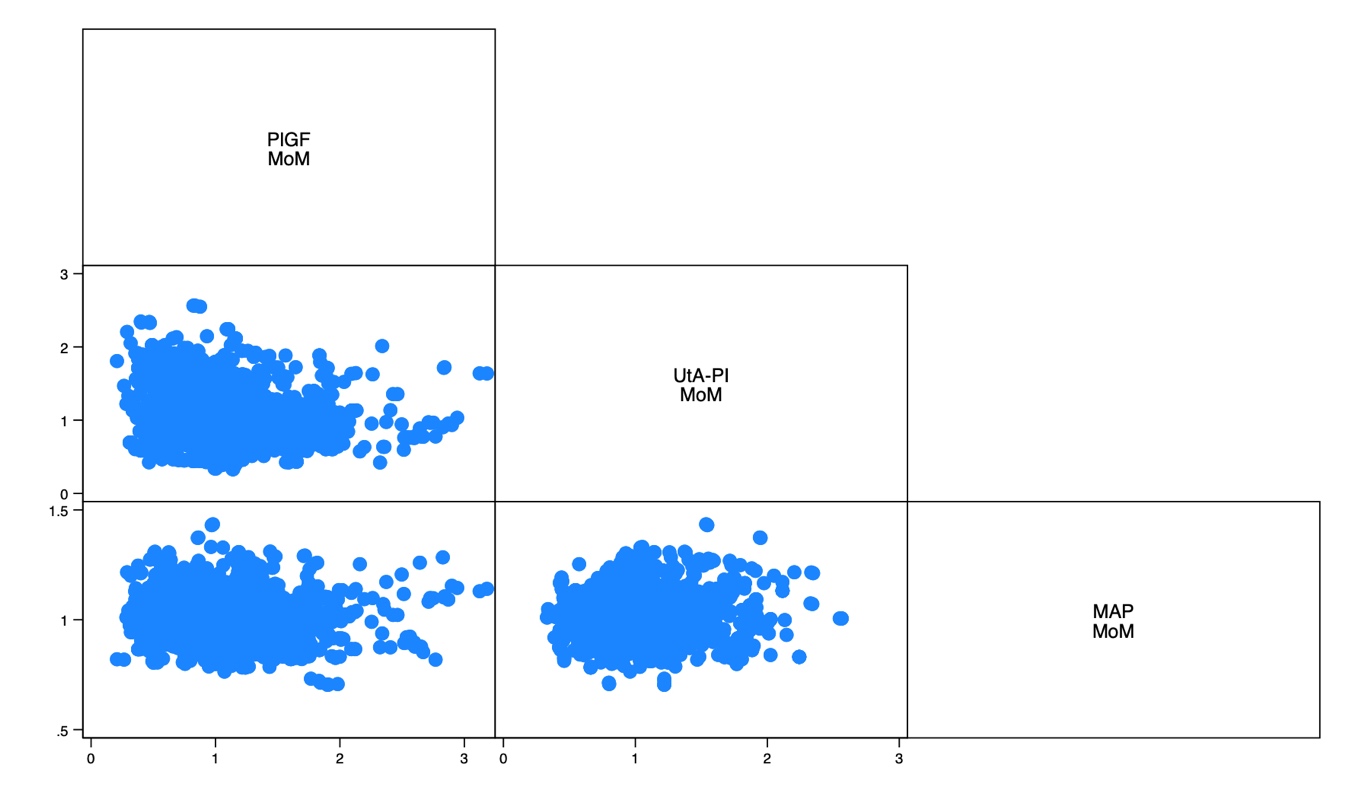
**

**Figure S1.** *Scatter matrix showing correlations between domains (MoM).* Pairwise scatter plots of MoM values for placental growth factor (PlGF mom), uterine artery pulsatility index (UtA mom), and mean arterial pressure (MAP mom). Weak pairwise correlations (|r| ≤ 0.13) support the partial independence and complementary physiopathologic information provided by each domain.


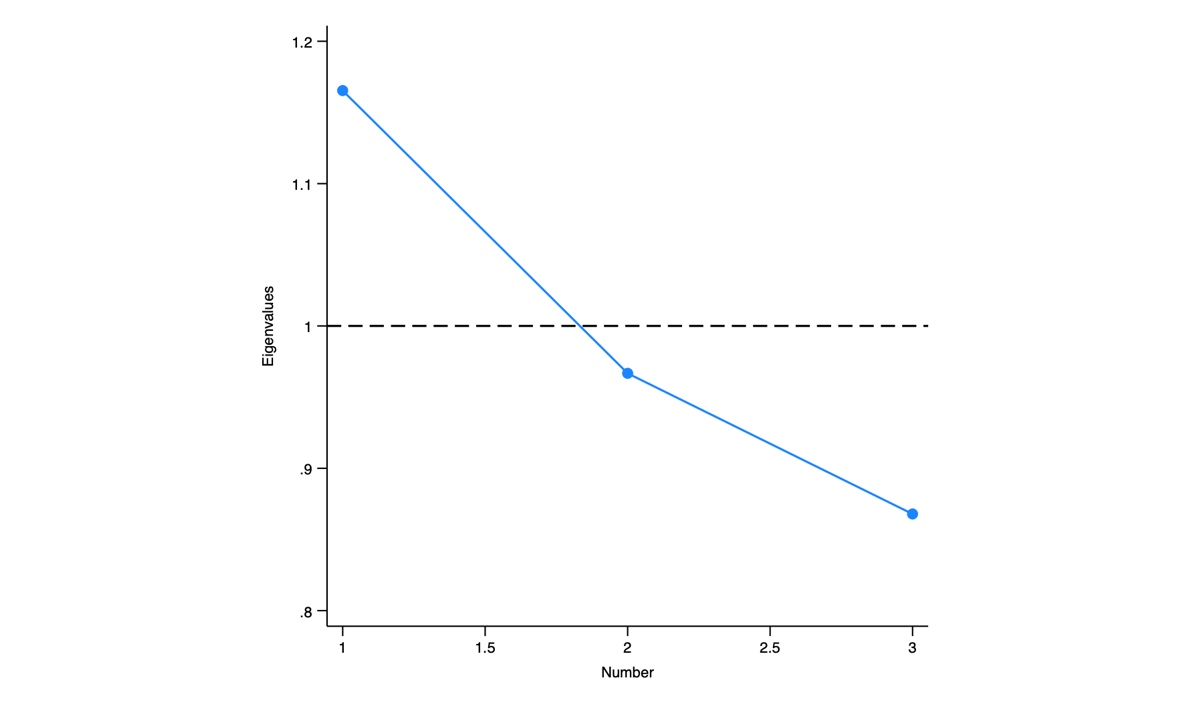

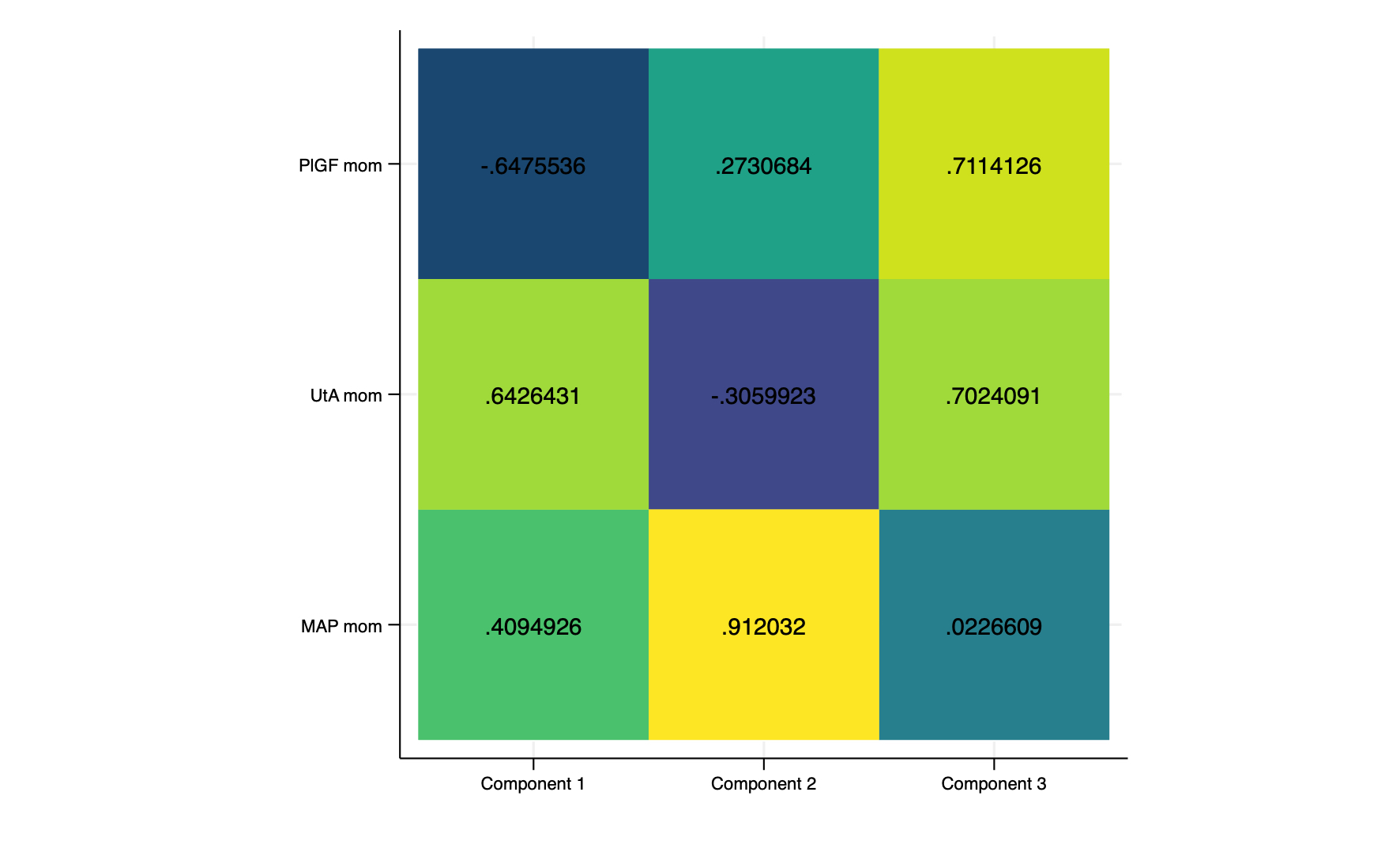


**Figure S2.** *Principal component analysis (PCA) of PlGF mom, UtA mom, and MAP mom.* *(A)* Scree plot of eigenvalues showing three principal components explaining 38.9%, 32.2%, and 28.9% of total variance (dashed line = Kaiser criterion, eigenvalue = 1). *(B)* Varimax-rotated component loadings demonstrating orthogonal axes, with near-exclusive loadings of UtA mom on Component 1 (uteroplacental domain), MAP mom on Component 2 (maternal-vascular domain), and PlGF mom on Component 3 (molecular/angiogenic domain).

**
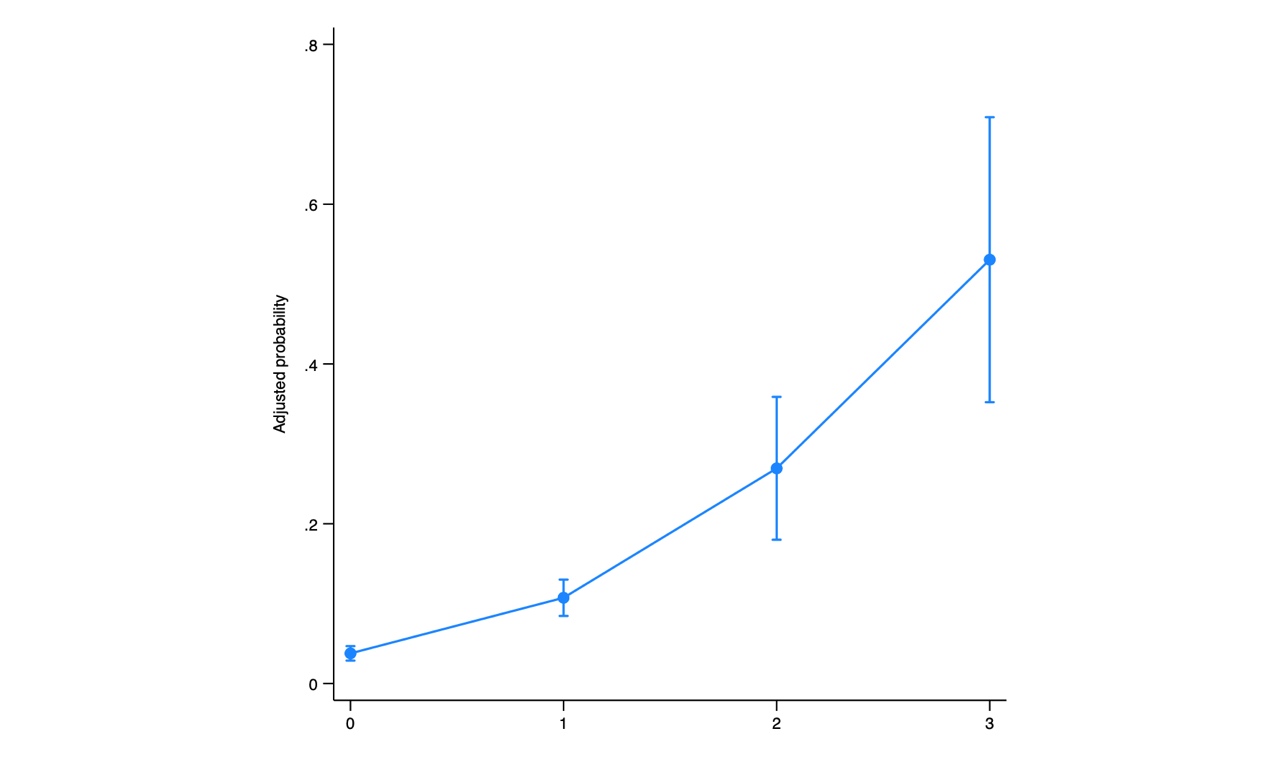
**

**Figure S3.** *Adjusted probability of preeclampsia (PE) according to the number of altered domains (0–3).* Logistic regression modeling of the cumulative number of physiopathologic abnormalities—molecular (PLGF p10), uteroplacental (UtA-PI p95), and maternal-tensional (MAP p95). The adjusted probability of PE increases almost exponentially from 3.8% (no abnormal domain) to 53.0% (all three altered), illustrating a continuous gradient in placental insufficiency severity.


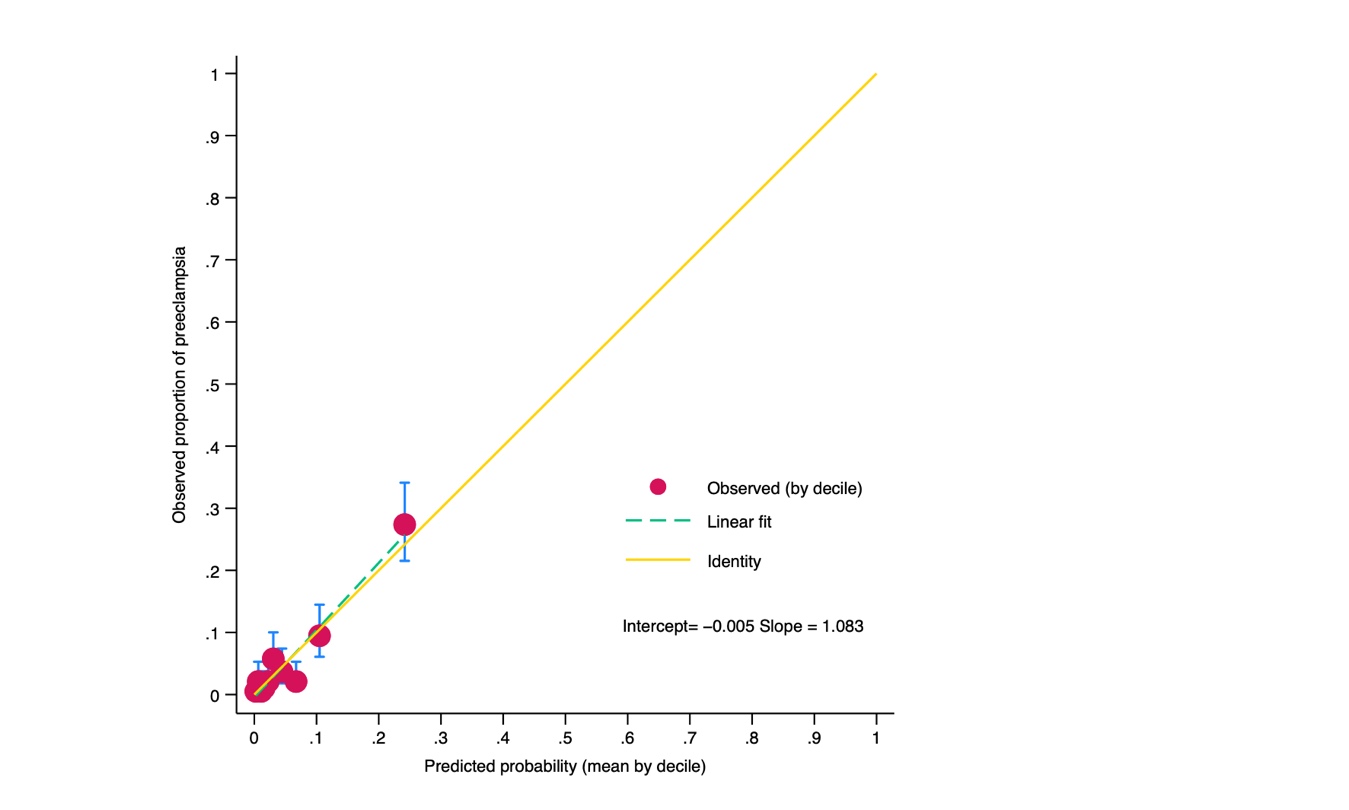


**Figure S4.** Calibration plot for Model D showing the agreement between predicted and observed probabilities of preeclampsia across deciles of risk. The solid yellow line represents perfect calibration (identity line), and the dashed green line indicates the fitted calibration slope (1.08). Points represent observed event rates (with 95% binomial confidence intervals). The calibration intercept was −0.005, confirming the absence of systematic bias.

**
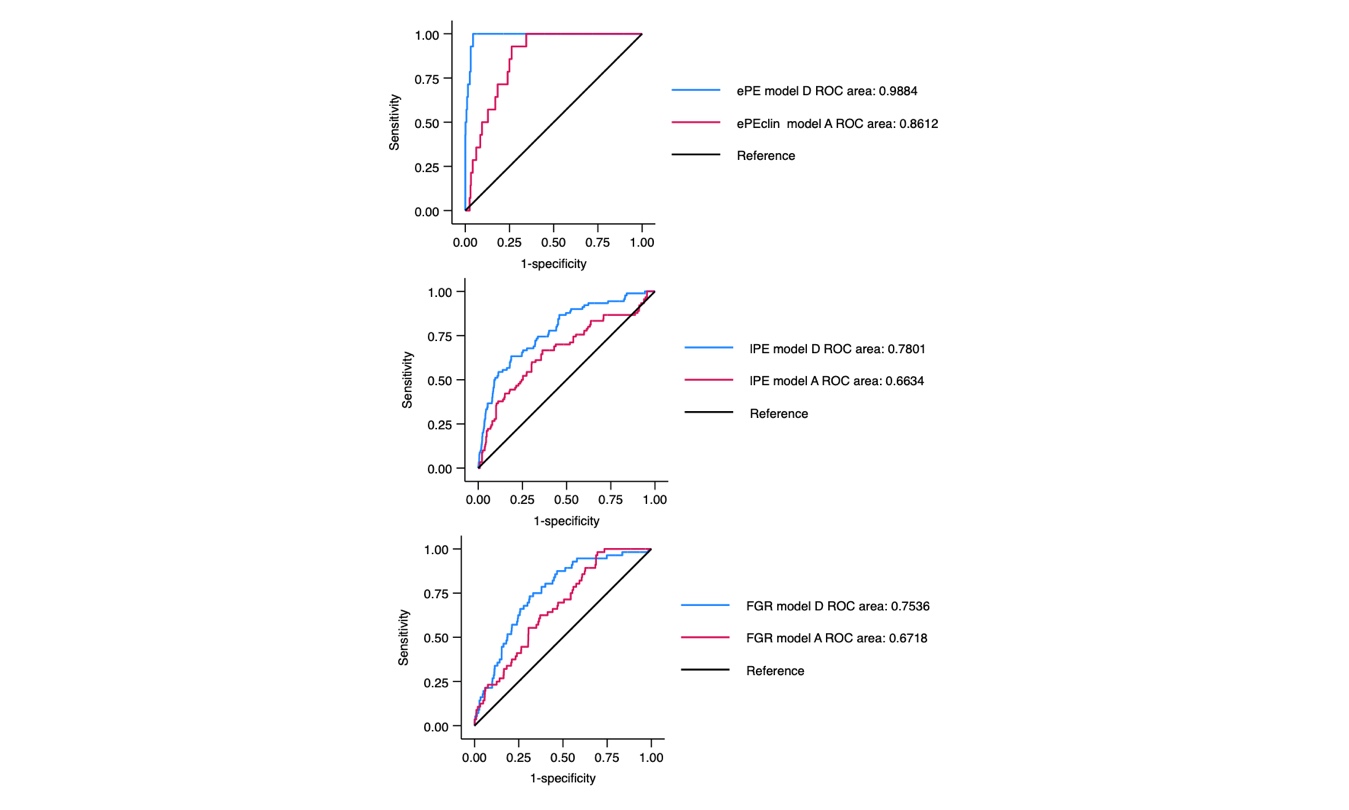
Figure S5.** Receiver-operating-characteristic (ROC) curves for the integrated three-domain model (Model D) and the baseline clinical model (Model A) in early-onset preeclampsia, late-onset preeclampsia, and isolated fetal growth restriction.
